# Supplementary material for: To address surface reaction network complexity using scaling relations machine learning and DFT calculations
Source: Nat Commun. 2017 Mar 6;8:14621. doi: 10.1038/ncomms14621 (PMC5343483; doi:10.1038/ncomms14621)
Supplement: Supplementary Information — Supplementary Figures, Supplementary Notes, Supplementary Methods and Supplementary References [file ncomms14621-s1.pdf]

Supplementary Methods

All surfaces are treated using the periodic slab approach with 20 Å of vacuum between slabs and a dipole correction is applied [1]. A slab size of 3x3x4 (k-point sampling 4x4x1) is used, resulting in adsorbate coverages of 1/9 ML and minimizing adsorbate-adsorbate interactions. In all calculations the lower 2 atomic layers were constrained to the bulk positions while the upper 2 layers were allowed to relax. Geometry relaxations were performed using a quasi-Newton algorithm as implemented in the Atomic Simulation Environment (ASE) [2] until the maximum force is less than 0.05 eV/Å. Multiple initial guesses were used for each adsorbate in an attempt to ensure that the geometries obtained are global minima. The initial guesses were generated by placing adsorbates at high-symmetry positions and by using a minima-hopping algorithm [3] within ASE in conjunction with the Hotbit Density Functional Tight Binding (DFTB) calculator [4]. The repulsion parameters for the DFTB calculator were parameterized using BEEF-vdW, and the minima-hopping algorithm was run until 10 unique minima were obtained for each adsorbate; of these minima, the 3 with the lowest energy were used as initial guesses for geometry optimization with BEEF-vdW.

Transition-state energies were calculated using the climbing-image nudged elastic band (CINEB) algorithm [5]. The initial state is assumed to be the lowest-energy configuration of a given adsorbate, and the final state was found by stretching the bond to be broken and relaxing the system; a linear interpolation between the two states was used as an initial guess. The CINEB spring constant used varies from system to system, but is generally on the order of 0.5 eV/Å, and the CINEB algorithm was run until the maximum force was less than 0.05 eV/Å.

The energies of surface intermediates and transition-states are reported as formation energies calculated by:

$$E_f = E_{slab+ads} - E_{slab} - \sum_{i \in \{C,H,O\}} n_i \mu_i \tag{1}$$

where $E_{slab+ads}$ is the raw DFT energy of the surface and adsorbate, E_{slab} is the raw DFT energy of the surface slab, n_i is the number of atoms of species i in the adsorbate, and μ_i are reference energies. In this case the reference energies are given by electronic potential energies (as opposed to Gibbs free energies) and are computed by:

$$\begin{aligned} \mu_O &= E_{H_2O} - E_{H_2} \\ \mu_C &= E_{CO} - \mu_O \\ \mu_H &= E_{H_2}/2 \end{aligned} \tag{2}$$

where E_j is the DFT gas-phase energy of species j. We applied corrections to gas-phase and surface adsorbates for species with known inaccuracies in descriptions by BEEF-vdW[6]. Corrections include gas-phase H_2 (+0.09 eV), Formic acid (+0.20 eV), CO_2 (+0.41 eV) and surface adsorbate COOH* (+0.20 eV). Binding energy correction for surface adsorbed species due to hydrogen-bond stabilization were included for OH* (-0.5 eV) and COOH* (-0.25eV).

Vibrational frequencies are calculated with a normal mode analysis by using a finite-difference approximation of the Hessian matrix as implemented in ASE. The finite difference delta is 0.01 Å with 4 displacements per Cartesian coordinate. Imaginary frequencies are assumed to correspond to very low vibrational modes and are replaced by 6.8 meV due to the fact that the entropy associated with a mode diverges as the mode goes to zero. The cutoff of 6.8 meV corresponds to an entropy of ca. $3k_B$ at 600 K; it is assumed that below this frequency the entropy would be bounded by other effects.

Gibbs free energies of adsorbed species are calculated using the harmonic approximation (all degrees of freedom are assumed to be vibrational and free energies are calculated from the harmonic oscillator partition function). Gas-phase free energies are computed using the ideal gas approximation with vibrational modes calculated using DFT, and the rotational moments of inertia are calculated using the DFT optimized geometries. It was verified that the ideal gas approximation agrees with tabulated free energies to within 0.05 eV for small molecules. All free energies are calculated at 573 K with 1 atm partial pressure for each of the gas phase species (CO, H_2, ethanol).

Supplementary Note 1: Reaction Mechanism Enumeration

The reaction network for intermediates with up to two carbons (C1/C2 chemistries) was built by constructing a list of elementary reaction steps and searching paths that connected the desired reactants and products. First, a list of C1 and C2 intermediates was constructed. All C1 intermediates were enumerated (e.g. CH_4, CH_3, CH_3OH). C2 intermediates with CCO or OCCO backbones were also included. Adding other intermediates of interest such as elemental species (H,O,C), H_2O, and OH resulted in a total of 99 intermediates of interest. Gas phase species of interest were also added, including CH_3CH_2OH, CH_3OH, CH_4, H_2O, H_2, CO_2, CH_3CHO, CO. Elementary reaction steps were then generated by including adsorption reactions for gas-phase species and generating all reactions that contained two adsorbed reactants and produced a valid intermediate (e.g. no C3 or above products). This process resulted in approximately 190 elementary reactions. Finally, pathways through the reaction network were generated by starting with the initial reactants, and performing a recursive search. The search was truncated at 8 reaction steps, and the resulting pathways were added if they reached one of the desired gas-phase final products. This recursive algorithm for mechanism enumeration is sufficient for networks of the size in this study, but the time complexity with respect to the number of reactions could be improved with more complex methods [7].

Supplementary Note 2: Prediction of Surface Intermediate Formation Energies

Formation free energies of intermediate surface species are predicted using a Gaussian process (GP) regression scheme with group-additivity based fingerprints as illustrated in Supplementary Figure 1. Given the chemical structure of an intermediate of interest, we first decompose the system into fragments using Extended Connectivity Fingerprints (ECFP) with radius-1 [8]. Each fragment represents the local environment of each atom by considering all atoms directly bonded. Bond types are used in the fingerprint. With only radius-1 fragments, there is one fragment per atom. Unlike other approaches that have aimed to develop the most accurate group-additivity approach for predicting these formation energies [9, 10], we explicitly do not include bonds to the surface since doing so generally requires some insight or prediction into precisely how the molecule is adsorbed onto the surface. Instead, this same physical insight is effectively included in the regression scheme. This allows this approach to be very flexible in quickly building networks for various surface species where the precise adsorption configuration is not initially known, but limits the transferability of the model between facets or surface adsorption sites. It is expected that electronic-structure calculations represent the lowest-energy configuration for a given adsorption site, as the described DFT methodology provides.

In the network of reactions on Rh(111), there are approximately 100 species, and roughly 45 unique fragments in the EFCP fingerprints. The dimensionality of this parameter space is comparable to the number of intermediates and thus including all of these fragments in a fitting procedure would lead to overfitting. We know that many of these fragments do not consist of independent quantities, but are actually dependent and related as demonstrated by the wide applicability of linear scaling relations[11]. Instead, we take the fingerprint for each molecule (a vector of dimension approximately 50), and reduce the dimensionality to just 10 using principle component analysis (PCA). This dimensionality reduction was performed after each iteration of the exploration algorithm, so that the precise mapping of the initial to final fingerprint space is different at each iteration. This process is equivalent to re-constructing a new set of linear scaling relations at each iteration. The number of final dimensions after the PCA reduction (either 10 or 15) did not have a significant effect on the accuracy or convergence of the process, suggesting that there were sufficient degrees of freedom for the group additivity method, and is expected given the remarkably broad applicability of far simpler linear scaling relations. This number could instead be chosen in an automated fashion instead using

cross-validation. The PCA reduction was only applied after the number of molecules in the training set was greater than the final number of dimensions in the PCA.

A GP regression scheme was used for the final prediction of the formation free energy for each intermediate relative to the gas phase reference species (CO, H_2, H_2O). The reduced dimension fingerprint (from the PCA analysis) was used as the input vector, and the free energy of formation (G_f) as the observable. The GP was trained on the available DFT measurements and re-trained at each iteration as the free energy of requested intermediates were added from DFT calculations. DFT uncertainty was propagated through the GP by constructing a GP for each of the BEEF ensembles. Uncertainty associated with the GP itself was not propagated in this scheme, as it would have required a more sophisticated sampling scheme to properly include the effects from multiple independent sources of uncertainty. The GP was used only for prediction of unmeasured species; if the formation energy of a species had been calculated with DFT, the measured free energy was simply used. A second GP was trained and used for the prediction of enthalpic energies (E_f) in an equivalent fashion. Both Gaussian processes were constructed using identical settings in the python module sklearn, with a squared exponential correlation function, a constant regression scheme, $\theta_0 = 0.01$ and a nugget of 0.05^2 (representing an assumed uncertainty of 0.05 eV in the underlying DFT calculations. These parameters were chosen empirically to balance a GP that would be accurate near points in the training set while still yielding useful predictions for trial points.

Supplementary Note 3: Scaling Relations Using CatApp

Estimates of transition state enthalpies were provided with linear scaling relations. Scaling relations between the enthalpy of reaction and enthalpic activation energy were constructed using the CatApp database [12]. Two scaling relations were constructed: one for hydrogenation reactions, and one for all other reactions, as illustrated in Supplementary Figure 2. The resulting linear relation was of the standard form

$$E_a = \alpha \Delta E^{rxn} + \beta, \tag{3}$$

where α, β are fitted constants for each reaction type and the energy of the transition state relative to the reference state (gas phase enthalpies) was

$$E_{TS} = (\alpha \Delta E^{rxn} + \beta) + E_{IS} \tag{4}$$

where E_{IS} represents the combined enthalpies of reactants, also relative to the reference gas phase species (H_2O, CO, and H_2). The slope of the BEP relation α was taken from fitting the measurements in CatApp. The measurements in this work were observed to lie within uncertainty of the CatApp data, but were slightly below the CatApp trend line. A linear regression for the measured Rh(111) hydrogenation data (in the form above, $\alpha \Delta E^{rxn} + \beta$) using the same slope ($\alpha = 0.69$) resulted in an offset about 0.2 eV lower than the CatApp trend line ($\beta = 0.70$ eV vs $\beta = 0.95$ eV for CatApp data). A similar process for non-hydrogenation reactions resulted in a significantly lower trend line ($\beta = 1.00$ eV vs $\beta = 1.57$ eV for CatApp), suggesting that the non-hydrogenation reactions in this work (mainly C-O scission, C-C scission and adsorption/desorption), were not representative of all of the reactions included in the CatApp database. Also contributing to this discrepancy is the inclusion of van der Waals interactions in this work, which were not present in most of the calculations in the CatApp database.

Similar relations were used to construct an estimate of the transition state free energies. We assumed that the BEP relations for free energy followed the same form

$$G_a = \alpha^G \Delta G^{rxn} + \beta^G. \tag{5}$$

In the absence of a database of transition state free energies (analogous to CatApp with enthalpic information) to calculate α^G, β^G, we found that the linear scaling relations for enthalpies were reasonable approximations for the free energy linear scaling relations, as shown in Supplementary Figure 2. For both hydrogenations and non-hydrogenation classes of reactions, the linear fits were nearly identical as to the fits for enthalpy, with β about 0.05 eV lower in both cases. Using just two classifications represents a conservative estimate of the uncertainty in this process. Improved classification of reactions (more than just hydrogenation, non-hydrogenation used here) would likely lead to more accurate BEP relations but would require a larger database of surface reactions.

Supplementary Note 4: Identification of Rate Limiting Steps

Pathways through the reaction network were evaluated by the rate-limiting transition state along the reaction pathway. In transition state theory, the rate-limiting step for reaction pathways in series with intermediate energies

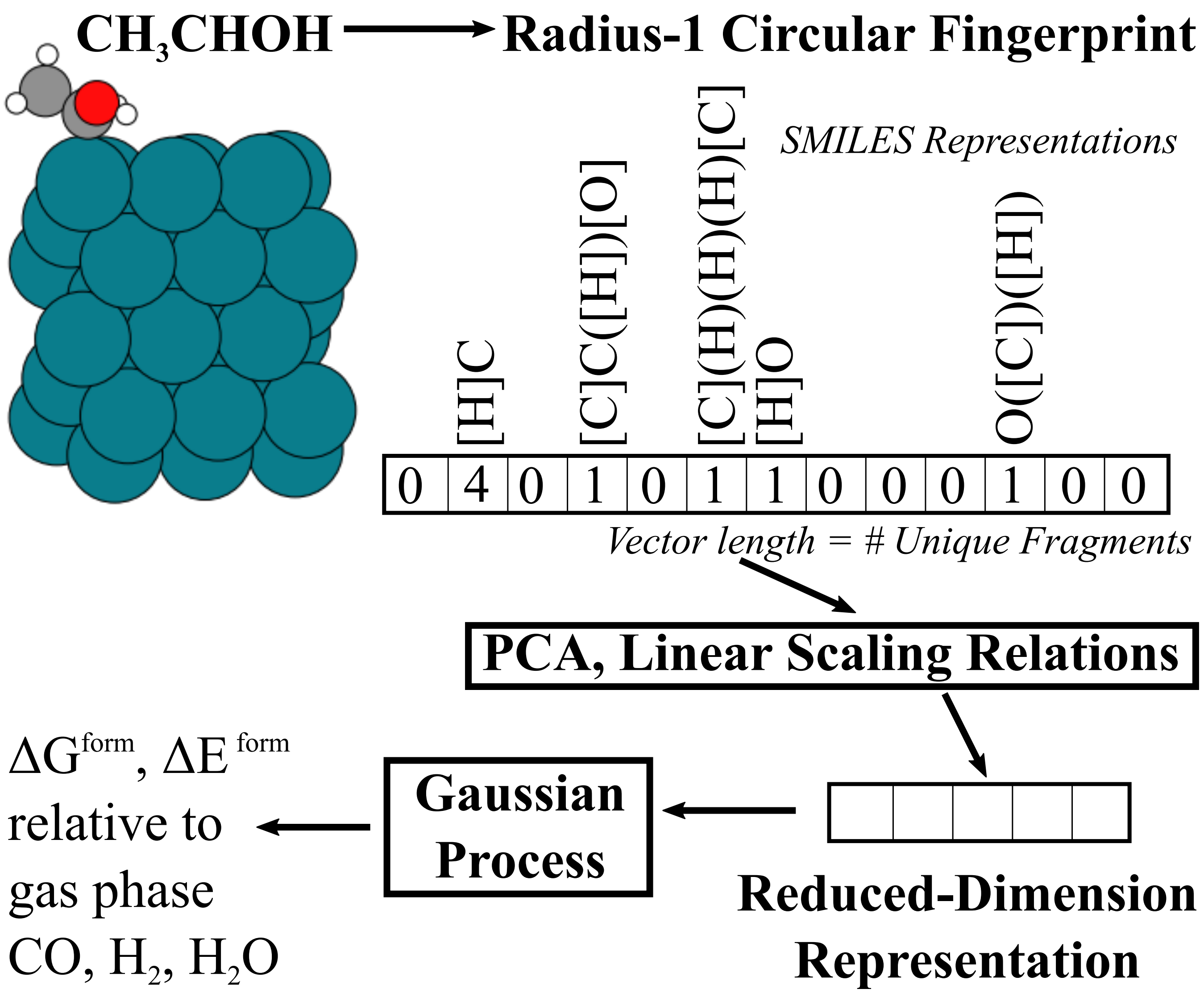

Supplementary Figure 1: Fingerprinting and regression scheme to generate estimates for formation energies of arbitrary small molecule surface adsorbates, demonstrated for the case of CH_3CHOH on Rh(111). Circular fingerprinting at radius-1 (1-bond radius environment for each atom) are used to generate fragments from adsorbate chemical structure and resulting fragment counts are stacked into a vector. This vector is reduced in dimension through principle component analysis, since fragment energies are not independent quantities. This final representation is used as input to a Gaussian Process (GP), which is trained on a number of measured formation energies with DFT.

all larger than the initial state will be reaction with the highest transition state free energy, $G_{\mathrm{TS}}^{\mathrm{max}}$, relative to the initial state. Pathways with varying sinks before the rate-limiting step would have different kinetics and require a more complicated treatment, such as the energetic-span approach from literature [13]. Pathways with smaller $G_{\mathrm{TS}}^{\mathrm{max}}$ were assumed to have higher fluxes through the rate-limiting steps. We ignore effects of coverage on transition state energies. With this simple criterion, we were then able to rapidly identify the likelihood of each reaction being the most-likely reaction, without resorting to expensive and more computationally demanding microkinetic models of the entire reaction pathway. This will not hold for reactions that have intermediate energies substantially lower than the initial state. Given the proximity of the rate-limiting step to the initial species in this work, this is not a concern, but could be addressed for other systems with a more detailed microkinetic model.

The highest transition state for each pathway $G_{\mathrm{TS}}^{\mathrm{max}}$ was evaluated using the predicted G_{TS} and corresponding uncertainty for each transition state in the pathway as outlined above. Uncertainty in the predicted G_{TS} for each species from underlying BEEF-vdW calculations and linear scaling relations were propagated by generating an ensemble of possible transition state values for each reaction. For each of the 2,000 parameterizations in the BEEF-vdW ensemble, 100 samples of possible linear scaling relation errors ($2{,}000 \cdot 100 = 200{,}000$ total samples) $G_{\mathrm{TS}}^{\mathrm{max}}$ was generated. For

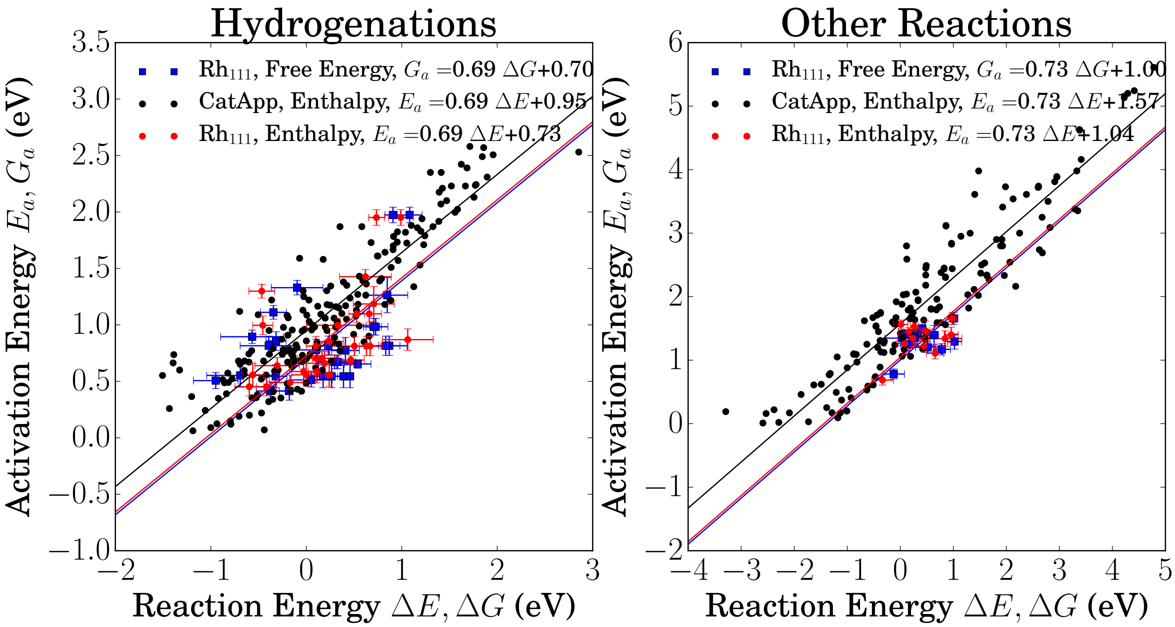

Supplementary Figure 2: Brønsted-Evans-Polanyi (BEP) relations used in this work. (left) BEP relations for hydrogen reactions. Black circles are entries in the CatApp database. Red circles are reaction enthalpies and activation energies (E_a) studied in this work. Blue circles are likewise reaction free energies ΔG and activation free energies G_a studied in this work. Linear regressions are included for each data set. (left) (right) All other reactions, including adsorption/desorption, C-C bond scission, and others. Activation energies for hydrogenation reactions are significantly lower than the other included reactions.

each of these samples, the pathway with the lowest $G_{\mathrm{TS}}^{\mathrm{max}}$ was evaluated. The probability of each path being the one with the lowest $G_{\mathrm{TS}}^{\mathrm{max}}$ was evaluated by counting the number of samples for which this was the case. This process allowed for correlations in the uncertainty of different species in the BEEF-vdW ensemble to propagate through to the end result. For example, uncertainty in the relative $G_{\mathrm{TS}}^{\mathrm{max}}$ for two pathways with the same rate-limiting step is substantially lower than for pathways with different rate-limiting steps. Reduced networks were then constructed by including pathways with progressively less likelihood of containing a lower $G_{\mathrm{TS}}^{\mathrm{max}}$ until until a desired probability of completeness was accumulated.

Supplementary Note 5: Final Reduced Mechanism at Reaction Conditions

Included is the most likely pathway to ethanol on Rh(111) at the given conditions according to first-principles DFT, as illustrated in Figure 3 of the main text. Reaction numbers correspond to the full list of elementary steps in the final section of the SI.

$$H* + H* \rightarrow H_2 \quad (0)$$
$$CO + * \rightarrow CO* \quad (1)$$
$$H_2O + * \rightarrow H_2O* \quad (2)$$
$$CH_3CH_2OH + * \rightarrow CH_3CH_2OH* \quad (6)$$
$$H* + OH* \rightarrow H_2O* \quad (9)$$
$$H* + CH* \rightarrow CH_2* \quad (11)$$
$$H* + CO* \rightarrow CHO* \quad (14)$$
$$H* + CO* \rightarrow COH* \quad (15)$$
$$H* + CH_2CHO* \rightarrow CH_3CHO* \quad (41)$$
$$H* + COH* \rightarrow CHOH* \quad (89)$$
$$H* + CH_3CHO* \rightarrow CH_3CH_2O* \quad (111)$$
$$OH* + CH* \rightarrow CHOH* \quad (169)$$
$$CH_2* + CHO* \rightarrow CH_2CHO* \quad (222)$$

Supplementary Note 6: List of Elementary Reaction Steps Considered

$$H* + H* \rightarrow H_2 \quad (0)$$
$$CO + * \rightarrow CO* \quad (1)$$
$$H_2O + * \rightarrow H_2O* \quad (2)$$
$$CH_4 + * \rightarrow CH_4* \quad (3)$$
$$CH_3OH + * \rightarrow CH_3OH* \quad (4)$$
$$CO_2 + * \rightarrow CO_2* \quad (5)$$
$$CH_3CH_2OH + * \rightarrow CH_3CH_2OH* \quad (6)$$
$$CH_3CHO + * \rightarrow CH_3CHO* \quad (7)$$
$$H* + O* \rightarrow OH* \quad (8)$$
$$H* + OH* \rightarrow H_2O* \quad (9)$$
$$H* + C* \rightarrow CH* \quad (10)$$
$$H* + CH* \rightarrow CH_2* \quad (11)$$
$$H* + CH_2* \rightarrow CH_3* \quad (12)$$
$$H* + CH_3* \rightarrow CH_4* \quad (13)$$
$$H* + CO* \rightarrow CHO* \quad (14)$$
$$H* + CO* \rightarrow COH* \quad (15)$$
$$H* + CHO* \rightarrow CH_2O* \quad (16)$$
$$H* + CHO* \rightarrow CHOH* \quad (17)$$
$$H* + CH_2O* \rightarrow CH_3O* \quad (18)$$
$$H* + CH_2O* \rightarrow CH_2OH* \quad (19)$$
$$H* + CO_2* \rightarrow HCOO* \quad (20)$$
$$H* + CO_2* \rightarrow COOH* \quad (21)$$
$$H* + COOH* \rightarrow HCOOH* \quad (22)$$
$$H* + COOH* \rightarrow HOCOH* \quad (23)$$
$$H* + HCOO* \rightarrow CH_2OO* \quad (24)$$
$$H* + CH_2OO* \rightarrow CH_2OOH* \quad (25)$$
$$H* + CCO* \rightarrow CHCO* \quad (26)$$
$$H* + CCO* \rightarrow CCHO* \quad (27)$$
$$H* + CCO* \rightarrow CCOH* \quad (28)$$
$$H* + CCHO* \rightarrow CHCHO* \quad (29)$$
$$H* + CCHO* \rightarrow CCH_2O* \quad (30)$$
$$H* + CCHO* \rightarrow CCHOH* \quad (31)$$
$$H* + CCOH* \rightarrow CHCOH* \quad (32)$$
$$H* + CHCO* \rightarrow CH_2CO* \quad (33)$$
$$H* + CCH_2O* \rightarrow CHCH_2O* \quad (34)$$
$$H* + CCH_2O* \rightarrow CCH_2OH* \quad (35)$$
$$H* + CCHOH* \rightarrow CHCHOH* \quad (36)$$
$$H* + CH_2CO* \rightarrow CH_3CO* \quad (37)$$
$$H* + CH_2CO* \rightarrow CH_2CHO* \quad (38)$$
$$H* + CH_2CO* \rightarrow CH_2COH* \quad (39)$$
$$H* + CCH_2OH* \rightarrow CHCH_2OH* \quad (40)$$
$$H* + CH_2CHO* \rightarrow CH_3CHO* \quad (41)$$
$$H* + CH_2CHO* \rightarrow CH_2CH_2O* \quad (42)$$
$$H* + CH_2CHO* \rightarrow CH_2CHOH* \quad (43)$$
$$H* + CH_2COH* \rightarrow CH_3COH* \quad (44)$$
$$H* + CH_2CH_2O* \rightarrow CH_3CH_2O* \quad (45)$$
$$H* + CH_2CH_2O* \rightarrow CH_2CH_2OH* \quad (46)$$
$$H* + CH_2CHOH* \rightarrow CH_3CHOH* \quad (47)$$
$$H* + CCOO* \rightarrow CHCOO* \quad (48)$$
$$H* + CCOO* \rightarrow CCOOH* \quad (49)$$
$$H* + CCOOH* \rightarrow CHCOOH* \quad (50)$$
$$H* + CCOOH* \rightarrow CCHOOH* \quad (51)$$
$$H* + CHCOO* \rightarrow CH_2COO* \quad (52)$$
$$H* + OCCHO* \rightarrow HOCCHO* \quad (53)$$
$$H* + OCCHO* \rightarrow OCHCHO* \quad (54)$$
$$H* + OCCHO* \rightarrow COCH_2O* \quad (55)$$
$$H* + OCCHO* \rightarrow OCCHOH* \quad (56)$$
$$H* + OCCOH* \rightarrow HOCCOH* \quad (57)$$
$$H* + OCCOH* \rightarrow OCHCOH* \quad (58)$$
$$H* + OCCOH* \rightarrow OCCHOH* \quad (59)$$
$$H* + CCHOOH* \rightarrow CHCHOOH* \quad (60)$$

$$H * + CH_2COO* \rightarrow CH_3COO* \quad (61)$$
$$H * + CH_2COO* \rightarrow CH_2CHOO* \quad (62)$$
$$H * + CH_2COO* \rightarrow CH_2COOH* \quad (63)$$
$$H * + COCH_2O* \rightarrow HOCCH_2O* \quad (64)$$
$$H * + COCH_2O* \rightarrow OCCH_2OH* \quad (65)$$
$$H * + HOCCHO* \rightarrow HOCCHOH* \quad (66)$$
$$H * + OCCH_2O* \rightarrow HOCCH_2O* \quad (67)$$
$$H * + OCCHOH* \rightarrow OCHCHOH* \quad (68)$$
$$H * + OCHCHO* \rightarrow OCH_2CHO* \quad (69)$$
$$H * + CH_2CHOO* \rightarrow CH_3CHOO* \quad (70)$$
$$H * + CH_2CHOO* \rightarrow CH_2CHOOH* \quad (71)$$
$$H * + CH_2COOH* \rightarrow CH_3COOH* \quad (72)$$
$$H * + HOCCH_2O* \rightarrow HOCCH_2OH* \quad (73)$$
$$H * + HOCCHOH* \rightarrow HOCHCHOH* \quad (74)$$
$$H * + OCCH_2OH* \rightarrow OCHCH_2OH* \quad (75)$$
$$H * + OCH_2CHO* \rightarrow OCH_2CH_2O* \quad (76)$$
$$H * + OCH_2CHO* \rightarrow OCH_2CHOH* \quad (77)$$
$$H * + CH_2CHOOH* \rightarrow CH_3CHOOH* \quad (78)$$
$$H * + HOCHCHOH* \rightarrow HOCH_2CHOH* \quad (79)$$
$$H * + OCH_2CH_2O* \rightarrow OCH_2CH_2OH* \quad (80)$$
$$H * + HOCH_2CHOH* \rightarrow HOCH_2CH_2OH* \quad (81)$$
$$O * + CCO* \rightarrow CCOO* \quad (82)$$
$$O * + CCHO* \rightarrow OCCHO* \quad (83)$$
$$H * + CHCOH* \rightarrow CH_2COH* \quad (84)$$
$$H * + CHCOH* \rightarrow CHCHOH* \quad (85)$$
$$H * + CH_3O* \rightarrow CH_3OH* \quad (86)$$
$$H * + CHCO* \rightarrow CHCHO* \quad (87)$$
$$H * + CHCO* \rightarrow CHCOH* \quad (88)$$
$$H * + COH* \rightarrow CHOH* \quad (89)$$
$$H * + HCOO* \rightarrow HCOOH* \quad (90)$$
$$H * + CH_2COH* \rightarrow CH_2CHOH* \quad (91)$$
$$H * + CH_3CO* \rightarrow CH_3CHO* \quad (92)$$
$$H * + CH_3CO* \rightarrow CH_3COH* \quad (93)$$
$$H * + CHCH_2O* \rightarrow CH_2CH_2O* \quad (94)$$
$$H * + CHCOOH* \rightarrow CH_2COOH* \quad (95)$$
$$H * + CHCOOH* \rightarrow CHCHOOH* \quad (96)$$
$$H * + CHCH_2O* \rightarrow CHCH_2OH* \quad (97)$$
$$H * + CHCHOH* \rightarrow CH_2CHOH* \quad (98)$$
$$H * + HOCCHO* \rightarrow HOCCH_2O* \quad (99)$$
$$H * + CHCHOH* \rightarrow CHCH_2OH* \quad (100)$$
$$H * + HOCCOH* \rightarrow HOCCHOH* \quad (101)$$
$$H * + CHOH* \rightarrow CH_2OH* \quad (102)$$
$$H * + OCCH_2O* \rightarrow OCCH_2OH* \quad (103)$$
$$H * + OCCHOH* \rightarrow HOCCHOH* \quad (104)$$
$$H * + CCHOH* \rightarrow CCH_2OH* \quad (105)$$
$$H * + OCCHOH* \rightarrow OCCH_2OH* \quad (106)$$
$$H * + HCOOH* \rightarrow CH_2OOH* \quad (107)$$
$$H * + OCHCHO* \rightarrow OCHCHOH* \quad (108)$$
$$H * + OCHCOH* \rightarrow OCHCHOH* \quad (109)$$
$$H * + CH_2CHOH* \rightarrow CH_2CH_2OH* \quad (110)$$
$$H * + CH_3CHO* \rightarrow CH_3CH_2O* \quad (111)$$
$$H * + CH_3CHO* \rightarrow CH_3CHOH* \quad (112)$$
$$H * + CH_2COOH* \rightarrow CH_2CHOOH* \quad (113)$$
$$H * + CH_3COO* \rightarrow CH_3CHOO* \quad (114)$$
$$H * + CH_3COO* \rightarrow CH_3COOH* \quad (115)$$
$$H * + CHCHOOH* \rightarrow CH_2CHOOH* \quad (116)$$
$$H * + CH_3COH* \rightarrow CH_3CHOH* \quad (117)$$
$$H * + CHCH_2OH* \rightarrow CH_2CH_2OH* \quad (118)$$
$$H * + HOCCHOH* \rightarrow HOCCH_2OH* \quad (119)$$
$$H * + OCCH_2OH* \rightarrow HOCCH_2OH* \quad (120)$$
$$H * + CH_2CH_2OH* \rightarrow CH_3CH_2OH* \quad (121)$$
$$H * + CH_3CH_2O* \rightarrow CH_3CH_2OH* \quad (122)$$
$$H * + CH_3CHOH* \rightarrow CH_3CH_2OH* \quad (123)$$
$$H * + OCHCHOH* \rightarrow HOCHCHOH* \quad (124)$$

$$H * + OCHCHOH* \rightarrow OCH_2CHOH* \quad (125)$$
$$H * + OCHCHOH* \rightarrow OCHCH_2OH* \quad (126)$$
$$H * + CH_2OH* \rightarrow CH_3OH* \quad (127)$$
$$H * + CH_3CHOO* \rightarrow CH_3CHOOH* \quad (128)$$
$$H * + CH_3COOH* \rightarrow CH_3CHOOH* \quad (129)$$
$$H * + CCOH* \rightarrow CCHOH* \quad (130)$$
$$H * + CHCHO* \rightarrow CH_2CHO* \quad (131)$$
$$H * + OCH_2CHOH* \rightarrow HOCH_2CHOH* \quad (132)$$
$$H * + OCH_2CHOH* \rightarrow OCH_2CH_2OH* \quad (133)$$
$$H * + OCHCH_2OH* \rightarrow OCH_2CH_2OH* \quad (134)$$
$$H * + CHCHO* \rightarrow CHCH_2O* \quad (135)$$
$$H * + OCH_2CH_2OH* \rightarrow HOCH_2CH_2OH* \quad (136)$$
$$O * + C* \rightarrow CO* \quad (137)$$
$$O * + CH* \rightarrow CHO* \quad (138)$$
$$O * + CH_2* \rightarrow CH_2O* \quad (139)$$
$$O * + CH_3* \rightarrow CH_3O* \quad (140)$$
$$O * + CO* \rightarrow CO_2* \quad (141)$$
$$O * + CHO* \rightarrow HCOO* \quad (142)$$
$$O * + CH_2O* \rightarrow CH_2OO* \quad (143)$$
$$H * + CHCHO* \rightarrow CHCHOH* \quad (144)$$
$$H * + CHCOO* \rightarrow CHCOOH* \quad (145)$$
$$O * + CCOH* \rightarrow OCCOH* \quad (146)$$
$$O * + CHCO* \rightarrow CHCOO* \quad (147)$$
$$O * + CHCO* \rightarrow OCCHO* \quad (148)$$
$$O * + CCH_2O* \rightarrow COCH_2O* \quad (149)$$
$$O * + CCHOH* \rightarrow OCCHOH* \quad (150)$$
$$O * + CH_2CO* \rightarrow CH_2COO* \quad (151)$$
$$O * + CH_2CO* \rightarrow COCH_2O* \quad (152)$$
$$O * + CHCHO* \rightarrow OCHCHO* \quad (153)$$
$$O * + CHCOH* \rightarrow OCHCOH* \quad (154)$$
$$O * + CHCOH* \rightarrow HOCCHO* \quad (155)$$
$$O * + CCH_2OH* \rightarrow OCCH_2OH* \quad (156)$$
$$O * + CH_2CHO* \rightarrow OCH_2CHO* \quad (157)$$
$$O * + CH_2CHO* \rightarrow CH_2CHOO* \quad (158)$$
$$O * + CH_2COH* \rightarrow HOCCH_2O* \quad (159)$$
$$O * + CH_3CO* \rightarrow CH_3COO* \quad (160)$$
$$O * + CHCH_2O* \rightarrow OCH_2CHO* \quad (161)$$
$$O * + CHCHOH* \rightarrow OCHCHOH* \quad (162)$$
$$O * + CH_2CH_2O* \rightarrow OCH_2CH_2O* \quad (163)$$
$$O * + CH_2CHOH* \rightarrow OCH_2CHOH* \quad (164)$$
$$O * + CH_3CHO* \rightarrow CH_3CHOO* \quad (165)$$
$$O * + CHCH_2OH* \rightarrow OCHCH_2OH* \quad (166)$$
$$O * + CH_2CH_2OH* \rightarrow OCH_2CH_2OH* \quad (167)$$
$$OH * + C* \rightarrow COH* \quad (168)$$
$$OH * + CH* \rightarrow CHOH* \quad (169)$$
$$OH * + CH_2* \rightarrow CH_2OH* \quad (170)$$
$$OH * + CH_3* \rightarrow CH_3OH* \quad (171)$$
$$OH * + CO* \rightarrow COOH* \quad (172)$$
$$OH * + CHO* \rightarrow HCOOH* \quad (173)$$
$$OH * + COH* \rightarrow HOCOH* \quad (174)$$
$$OH * + CH_2O* \rightarrow CH_2OOH* \quad (175)$$
$$OH * + CCO* \rightarrow CCOOH* \quad (176)$$
$$OH * + CCO* \rightarrow OCCOH* \quad (177)$$
$$OH * + CCHO* \rightarrow HOCCHO* \quad (178)$$
$$OH * + CCHO* \rightarrow CCHOOH* \quad (179)$$
$$OH * + CCHO* \rightarrow OCHCOH* \quad (180)$$
$$OH * + CCOH* \rightarrow HOCCOH* \quad (181)$$
$$OH * + CHCO* \rightarrow CHCOOH* \quad (182)$$
$$OH * + CHCO* \rightarrow OCCHOH* \quad (183)$$
$$OH * + CCH_2O* \rightarrow HOCCH_2O* \quad (184)$$
$$OH * + CCHOH* \rightarrow HOCCHOH* \quad (185)$$
$$OH * + CH_2CO* \rightarrow CH_2COOH* \quad (186)$$
$$OH * + CH_2CO* \rightarrow OCCH_2OH* \quad (187)$$
$$OH * + CHCHO* \rightarrow CHCHOOH* \quad (188)$$

$$OH* + CHCHO* \rightarrow OCHCHOH* \quad (189)$$
$$OH* + CHCOH* \rightarrow HOCCHOH* \quad (190)$$
$$OH* + CCH_2OH* \rightarrow HOCCH_2OH* \quad (191)$$
$$OH* + CH_2CHO* \rightarrow CH_2CHOOH* \quad (192)$$
$$OH* + CH_2CHO* \rightarrow OCHCH_2OH* \quad (193)$$
$$OH* + CH_2COH* \rightarrow HOCCH_2OH* \quad (194)$$
$$OH* + CH_3CO* \rightarrow CH_3COOH* \quad (195)$$
$$OH* + CHCH_2O* \rightarrow OCH_2CHOH* \quad (196)$$
$$OH* + CHCHOH* \rightarrow HOCHCHOH* \quad (197)$$
$$OH* + CH_2CH_2O* \rightarrow OCH_2CH_2OH* \quad (198)$$
$$OH* + CH_2CHOH* \rightarrow HOCH_2CHOH* \quad (199)$$
$$OH* + CH_3CHO* \rightarrow CH_3CHOOH* \quad (200)$$
$$OH* + CHCH_2OH* \rightarrow HOCH_2CHOH* \quad (201)$$
$$OH* + CH_2CH_2OH* \rightarrow HOCH_2CH_2OH* \quad (202)$$
$$C* + CO* \rightarrow CCO* \quad (203)$$
$$C* + CHO* \rightarrow CCHO* \quad (204)$$
$$C* + COH* \rightarrow CCOH* \quad (205)$$
$$C* + CH_2O* \rightarrow CCH_2O* \quad (206)$$
$$C* + CHOH* \rightarrow CCHOH* \quad (207)$$
$$C* + CH_2OH* \rightarrow CCH_2OH* \quad (208)$$
$$C* + CO_2* \rightarrow CCOO* \quad (209)$$
$$C* + COOH* \rightarrow CCOOH* \quad (210)$$
$$C* + HCOOH* \rightarrow CCHOOH* \quad (211)$$
$$CH* + CO* \rightarrow CHCO* \quad (212)$$
$$CH* + CHO* \rightarrow CHCHO* \quad (213)$$
$$CH* + COH* \rightarrow CHCOH* \quad (214)$$
$$CH* + CH_2O* \rightarrow CHCH_2O* \quad (215)$$
$$CH* + CHOH* \rightarrow CHCHOH* \quad (216)$$
$$CH* + CH_2OH* \rightarrow CHCH_2OH* \quad (217)$$
$$CH* + CO_2* \rightarrow CHCOO* \quad (218)$$
$$CH* + COOH* \rightarrow CHCOOH* \quad (219)$$
$$CH* + HCOOH* \rightarrow CHCHOOH* \quad (220)$$
$$CH_2* + CO* \rightarrow CH_2CO* \quad (221)$$
$$CH_2* + CHO* \rightarrow CH_2CHO* \quad (222)$$
$$CH_2* + COH* \rightarrow CH_2COH* \quad (223)$$
$$CH_2* + CH_2O* \rightarrow CH_2CH_2O* \quad (224)$$
$$CH_2* + CHOH* \rightarrow CH_2CHOH* \quad (225)$$
$$CH_2* + CH_2OH* \rightarrow CH_2CH_2OH* \quad (226)$$
$$CH_2* + CO_2* \rightarrow CH_2COO* \quad (227)$$
$$CH_2* + COOH* \rightarrow CH_2COOH* \quad (228)$$
$$CH_2* + HCOO* \rightarrow CH_2CHOO* \quad (229)$$
$$CH_2* + HCOOH* \rightarrow CH_2CHOOH* \quad (230)$$
$$CH_3* + CO* \rightarrow CH_3CO* \quad (231)$$
$$CH_3* + CHO* \rightarrow CH_3CHO* \quad (232)$$
$$CH_3* + COH* \rightarrow CH_3COH* \quad (233)$$
$$CH_3* + CH_2O* \rightarrow CH_3CH_2O* \quad (234)$$
$$CH_3* + CHOH* \rightarrow CH_3CHOH* \quad (235)$$
$$CH_3* + CH_2OH* \rightarrow CH_3CH_2OH* \quad (236)$$
$$CH_3* + CO_2* \rightarrow CH_3COO* \quad (237)$$
$$CH_3* + COOH* \rightarrow CH_3COOH* \quad (238)$$
$$CH_3* + HCOO* \rightarrow CH_3CHOO* \quad (239)$$
$$CH_3* + HCOOH* \rightarrow CH_3CHOOH* \quad (240)$$
$$CO* + CHO* \rightarrow OCCHO* \quad (241)$$
$$CO* + COH* \rightarrow OCCOH* \quad (242)$$
$$CO* + CH_2O* \rightarrow COCH_2O* \quad (243)$$
$$CO* + CHOH* \rightarrow OCCHOH* \quad (244)$$
$$CO* + CH_2OH* \rightarrow OCCH_2OH* \quad (245)$$
$$CHO* + COH* \rightarrow OCHCOH* \quad (246)$$
$$CHO* + COH* \rightarrow HOCCHO* \quad (247)$$
$$CHO* + CH_2O* \rightarrow OCH_2CHO* \quad (248)$$
$$CHO* + CHOH* \rightarrow OCHCHOH* \quad (249)$$
$$CHO* + CH_2OH* \rightarrow OCHCH_2OH* \quad (250)$$
$$COH* + CH_2O* \rightarrow HOCCH_2O* \quad (251)$$
$$COH* + CHOH* \rightarrow HOCCHOH* \quad (252)$$

$$COH* + CH_2OH* \rightarrow HOCCH_2OH* \quad (253)$$
$$CH_2O* + CHOH* \rightarrow OCH_2CHOH* \quad (254)$$
$$CH_2O* + CH_2OH* \rightarrow OCH_2CH_2OH* \quad (255)$$
$$CHOH* + CH_2OH* \rightarrow HOCH_2CHOH* \quad (256)$$

[1] Bengtsson, L. Dipole correction for surface supercell calculations. *Physical Review B* **59**, 12301–12304 (1999).
[2] Bahn, S. R. & Jacobsen, K. W. An object-oriented scripting interface to a legacy electronic structure code. *Comput. Sci. Eng.* **4**, 56–66 (2002).
[3] Goedecker, S. Minima hopping: An efficient search method for the global minimum of the potential energy surface of complex molecular systems. *J. Chem. Phys.* **120**, 9911–9917 (2004).
[4] Koskinen, P. & Mäkinen, V. Density-functional tight-binding for beginners. *Computational Materials Science* **47**, 237–253 (2009).
[5] Henkelman, G., Uberuaga, B. P. & Jonsson, H. A climbing image nudged elastic band method for finding saddle points and minimum energy paths. *J. Chem. Phys.* **113**, 9901–9904 (2000).
[6] Yoo, J. S., Christensen, R., Vegge, T., Nørskov, J. K. & Studt, F. Theoretical Insight into the Trends that Guide the Electrochemical Reduction of Carbon Dioxide to Formic Acid. *ChemSusChem* n/an/a (2015).
[7] Rangarajan, S., Kaminski, T., Van Wyk, E., Bhan, A. & Daoutidis, P. Language-oriented rule-based reaction network generation and analysis: Algorithms of ring. *Computers & Chemical Engineering* **64**, 124–137 (2014).
[8] Rogers, D. & Hahn, M. Extended-Connectivity Fingerprints. *J. Chem. Inf. Model.* **50**, 742–754 (2010).
[9] Vorotnikov, V., Wang, S. & Vlachos, D. G. Group Additivity for Estimating Thermochemical Properties of Furanic Compounds on Pd(111). *Ind. Eng. Chem. Res.* **53**, 11929–11938 (2014).
[10] Salciccioli, M., Chen, Y. & Vlachos, D. G. Density Functional Theory-Derived Group Additivity and Linear Scaling Methods for Prediction of Oxygenate Stability on Metal Catalysts: Adsorption of Open-Ring Alcohol and Polyol Dehydrogenation Intermediates on Pt-Based Metals. *The Journal of Physical Chemistry C* **114**, 20155–20166 (2010).
[11] Nørskov, J. K., Bligaard, T., Rossmeisl, J. & Christensen, C. H. Towards the computational design of solid catalysts. *Nature Chem* **1**, 37–46 (2009).
[12] Hummelshøj, J. S., Abild-Pedersen, F., Studt, F., Bligaard, T. & Nørskov, J. K. CatApp: A Web Application for Surface Chemistry and Heterogeneous Catalysis. *Angew. Chem.* **124**, 278–280 (2011).
[13] Kozuch, S. & Shaik, S. How to conceptualize catalytic cycles? the energetic span model. *Accounts of Chemical Research* **44**, 101–110 (2011).
